# OVERCOMING OLDER ADULT RESISTANCE TO TECHNOLOGY TO SUPPORT DIGITAL HEALTH LITERACY: A CBPR APPROACH

**DOI:** 10.1093/geroni/igac059.2705

**Published:** 2022-12-20

**Authors:** Paul Freddolino, Fei Sun, Ha-Neul Kim, Dona Wishart, Eileen Godek, Megan Bentley

**Affiliations:** Michigan State University, East Lansing, Michigan, United States; Michigan State University, East Lansing, Michigan, United States; Michigan State University, East Lansing, Michigan, United States; Administration, Otsego, Michigan, United States; Otsego County Commission on Aging, Otsego, Michigan, United States; Michigan State University, East Lansing, Michigan, United States

## Abstract

Overall low digital use by older adults creates a barrier to tools that enable them access to basic and specialty healthcare through telehealth and address their isolation and loneliness. In particular, older adults with low-income, like low-income isolated Home Delivered Meal (HDM) program recipients, can be most vulnerable to this barrier. A university team and a local Commission on Aging jointly secured funding to pilot the Virtual Table (VT) model which builds on relationships between HDM drivers and meal recipients to overcome lack of interest and skepticism of technology like the internet and video chatting. For the pilot, 25 recipients were recruited to learn basic technology skills (e.g., email, video chat) from trusted peer tutors through weekly one-to-one sessions, in-person or by video, leading to knowledge and skills to support telehealth utilization. Twenty recipients (80%) are completing the pilot. Results to date show increased technology use and comfort, together with confidence to use telehealth. Participants valued the interactions with tutors as well as what they learned. Feedback from tutors, agency staff, and participants has been used to refine the pilot during implementation. Building on the lessons learned and feedback, the goal for the next stage of collaboration is developing a set of effective tools to implement VT in several diverse communities. Four VT ‘graduates’ will be actively involved as co-producers of these revised tools. In addition, older adult home-delivered-meal (HDM) recipients, agency staff, tutors, and other older adult volunteers will directly participate in ongoing evaluation of the new resources.

